# Nurses' Ethics in the Care of Patients During the COVID-19 Pandemic

**DOI:** 10.3389/fmed.2021.589550

**Published:** 2021-05-07

**Authors:** Aladeen Alloubani, Wejdan Khater, Laila Akhu-Zaheya, Maysa Almomani, Safa Alashram

**Affiliations:** ^1^Nursing Research Unit, King Hussein Cancer Center, Amman, Jordan; ^2^Department of Adult Health Nursing, Faculty of Nursing, Jordan University of Science and Technology, Irbid, Jordan; ^3^Consultancy of Secretary General, Civil Service Bureau, Amman, Jordan

**Keywords:** ethical, COVID-19, nurses, dilemma approach, care

## Abstract

Working during an epidemic can be physically, emotionally, and morally demanding for nurses. In addition to caring for patients, nurses are also responsible for looking after themselves and their families. The current study aimed to explore nurses' ethics in the care of patients during the coronavirus disease 2019 (COVID-19) pandemic. A descriptive qualitative approach was adopted in order to gain an in-depth understanding of nurses' experiences of caring for patients with coronavirus. A purposive sample of 10 nurses working with patients with COVID-19 was recruited. Interviews were held with the nurses, and content analysis of the interviews was conducted. Each interview was transcribed, and the text was coded into manageable categories on the word, word sense, phrase, sentence, and theme levels before analysis. Three major themes related to the nurses' ethical commitments during the COVID-19 crisis emerged during the data analysis. These themes are as follows: the obligation of nurses to provide care for patients regardless of their medical diagnosis; the ethical dilemma faced by nurses of whether to care for patients or protect themselves from the virus; and finally, the responsibility of nurses to care for themselves.

## Introduction

Nurses have always played an essential role in the provision of healthcare (1, 2). However, particularly during disasters and pandemics, nurses are exposed to greater risk and are required to work to their full capacity under risky circumstances. Working during an epidemic can be particularly demanding for nurses. Furthermore, nurses may often find themselves faced with moral dilemmas when working during pandemics, as they must balance between caring for patients while looking after themselves and their families (3).

In situations where there are limited resources available, such as the lack of personal protective equipment for healthcare providers during the COVID-19 pandemic, nurses must place their lives at risk in order to provide patient care (4, 5). Thus, nurses may feel unsafe, exposed to higher risks, and in need of professional, legal, and moral support while providing care during emergencies and crises (6).

Three main ethics challenges are likely to affect nurses in distinct ways, including nurses' safety, patients, families, and friends; the distribution of scarce resources; and the change in nature of nurses' relationships with patients and families (7).

As per Interpretive Statement 8.4 provided within the Code of Ethics for Nurses with Interpretive Statements (2015), all necessary actions performed by nurses and avoidance of action by nurses in the context of patient care can bring about outcomes that may be an accidental violation of human rights (8). Nurses must practice caution when deciding whether or not to participate in patient care in every situation, and this requires them to analyze the pros and cons of the situation so that they may be able to justify their actions when required to do so.

## Aim

The current study aimed to explore nurses' ethics in the care of patients during the COVID-19 pandemic.

## Methods

### Design

A descriptive qualitative approach was used to gain an in-depth understanding of nurses' experiences of caring for patients with coronavirus. The descriptive qualitative approach used in this study focused on answering “who,” “what,” and “where” questions related to the ethical experiences of nurses caring for patients with coronavirus (9). Moreover, this study was highly concerned with capturing the experiences and feelings of the respondents, as well as identifying specific trends in the study participants and personal characteristics. The use of a descriptive qualitative approach ensures that the information obtained from the respondents complies with scientific requirements (10).

### Sample and Setting

A purposive sample of 10 nurses working with patients with COVID-19 was recruited. Purposive sampling allows researchers to decide what needs to be known and set out to find participants who can and are willing to provide the most relevant information by virtue of knowledge or experience (11). Hence, purposive sampling was viewed to be suited to the aim of understanding nurses' ethical behaviors, attitudes, and practices during the COVID-19 pandemic. The sample size was determined based on data saturation, which refers to “the repetition of discovered information and confirmation of previously collected data” [(12), p. 122].

The study was carried out at two different hospitals in Jordan, one located in Amman, the capital of Jordan, and the other in the North Region of Jordan. The two selected hospitals are government hospitals that were the only hospitals receiving patients with confirmed COVID-19 diagnosis at the time of data collection. The inclusion criteria included being a nurse who provided direct nursing care for patients with COVID-19 and agreeing to participate in the study. Meanwhile, nurses who were working at the selected hospitals but in units that were not receiving confirmed COVID-19 cases were excluded.

### Interview Outline

The interview questions were developed based on a review of relevant articles in the literature and experts' opinions. Semi-structured, open-ended interviews that lasted between 30 and 90 min each were held *via* Zoom with 10 nurses. The interviews were guided by an interview guideline, and all interviews were held in Arabic. The interviews were tape-recorded, transcribed verbatim in Arabic, translated into English, and then analyzed using thematic analysis. The transcripts were translated from Arabic into English by the research team and then checked by a qualified translator and one of the study participants, so as to ensure the highest level of accuracy.

### Data Collection

The study aim and significance were explained to the participants prior to data collection, and the interviews were scheduled at the participants' convenience. The data collection process was an iterative process that included collecting, coding, and analyzing data (13). The interviews were conducted by the research team members, most of whom had previous experience conducting qualitative interviews. One-on-one interviews were held through the software application Zoom, and all interviews were recorded and kept strictly private and confidential.

The participants were informed that they had the right to withdraw from the study at any time without consequences, and that all collected data would be kept confidential. During data collection, the researchers built rapport with the respondents but made sure not to interfere with or impact their responses. To enhance the data authenticity and avoid any bias, several strategies were employed by the researchers, including active listening, unconditional acceptance, and clarification. In order to increase the reliability of the results, the interviewer summarized each interview to the interviewee at the end in order to allow the participant to check for and clarify any misconceptions or add additional information.

### Content Analysis

Thematic analysis was used for analyzing the data. All audiotaped interviews were carefully transcribed verbatim. Manual analysis was performed by the research team through constant reading and rereading, coding, and analysis of the data collected from the participants. Each interview was analyzed using the same process until all transcripts had been analyzed. The process of coding, categorizing, and originating the major themes were discussed with all research team members to ensure accuracy and consistency.

Content analysis of the translated transcripts was conducted, whereby the transcripts were coded into manageable categories on the word, word sense, phrase, sentence, and theme levels and then examined using either conceptual analysis or relational analysis. The researchers cross-checked their interpretations to validate the accuracy of the findings. Finally, to ensure the credibility of the results, member checks were carried out, where researchers shared the study's results with the participants in order to confirm that the findings were reflective of their experiences (14).

### Ethical Consideration

Prior to conducting the study, ethical approval was obtained from the institutional review board of Jordan University of Science and Technology. In order to maintain their privacy, the participants were informed that the interviews would be recorded, and each participant was able to choose a convenient location for the interview to be held through Zoom. Since the interviews were held *via* Zoom, a waiver of documentation of informed consent was requested. Finally, all collected data were stored on a password-protected computer.

## Results

### Demographic Data

Fifteen participants were invited to participate, of whom 10 (66.6%) agreed to participate and were therefore interviewed (Table 1). Of the 10 nurses, six were male, and four were female. The mean age of the participants was 32.6 years (*R* = 26–52), and the average number of years of experience was 11.9 years. As for educational level, all of the nurses held Bachelor of Science in Nursing (BSN) degrees, and three of the nurses also held master's degrees. All of the participating nurses had started caring for patients with COVID-19 since the virus had started spreading in Jordan (March 2, 2020).

**Table 1:** Participants demographics characteristics.

**Participants (Nurse No code)**	**Gender**	**Age (year)**	**Education level**	**Total number of experiences (year)**
NH1	Male	35	BS	14
NH2	Female	33	BS	12
NH3	Female	34	Master	13
NH4	Male	32	BS	12
NH5	Male	52	BS	31
NK1	Male	26	Master	5
NK2	Female	32	BS	12
NK3	Male	30	Master	10
NK4	Male	26	BS	5
NK5	Female	26	BS	5

### Themes

Three major themes related to the nurses' ethical commitment during the COVID-19 pandemic emerged during the data analysis (Table 2). These themes are as follows: the obligation of nurses to provide care for patients regardless of their medical diagnosis, the ethical dilemma faced by nurses of whether to care for patients or protect themselves from the virus, and the responsibility of nurses to care for themselves.

**Table 2:** Themes and subthemes.

**Theme**	**Sub themes**
Nurses are obligated to provide care for patients regardless	We are always available for patients
	Patient with Covid-19 has the right to be cared for
	What if this patient one of my family members
Ethical dilemma	Nurses should not be forced: it should be voluntary
	It is community and professional commitment
Nurses are responsible to protect themselves	

### The Obligation of Nurses to Provide Care for Patients Regardless of Their Medical Diagnosis

This theme describes nurses' perception of their ethical commitment to provide care for patients regardless of their medical diagnoses. This theme included three subthemes: nurses should always be available for patients; patients with COVID-19 have the right to Be cared for; and patients should be treated as if they were family members.

#### Nurses Should Always Be Available for Patients

The nurses believed that it was their duty as nurses to care for patients regardless of their diagnoses. The nurses also expressed that they were committed to being available for their patients when they needed them and providing the best care possible. One participant said:

“*For me, it is my role as a nurse to take care of people”* (NK3).

Another participant emphasized the importance of providing nursing care to the best of one's ability:

“*You need to work with all you have, with humanity, and with passion, and to try to give 100%”* (NH2).

Although the nurses expressed facing several challenges in caring for patients with COVID-19, they believed that this was part of their responsibilities. One participant reported:

“*It* [nursing] *is a humanitarian profession; even if there are challenges, we need to give patients their rights…that is a must”* (NK3).

Another participant said:

“*It doesn't make sense for someone with my experiences to no help people, as this is my job. It's important that I'm sincere toward my job”* (NH10).

#### Patients With COVID-19 Have the Right to Be Cared for

The participating nurses highlighted that COVID-19 patients had the same right of being cared for as did other patients. They emphasized that patients had the right to receive the care they needed because being COVID-19 patients was not their fault. One participant said:

“*Whatever the case, they are not responsible for their disease; they need someone to take care of them… that's the idea”* (NH3).

The participating nurses also expressed that COVID-19 patients are like patients with any other infectious disease, and that the only difference is that COVID-19 is a new virus. Thus, the nurses believed that COVID-19 patients had the same right to receive nursing care as did other patients. One nurse said:

“*As nurses, what is our job? What is needed from us? It is our job to provide nursing care for all patients … After all, patients with COVID-19 are like other patients, except that they have a new disease”* (NK4).

Another participant indicated:

“*I mean, patients with coronavirus are like other patients, except that the virus they have been infected with is new. Since I now have experience dealing with the virus, I have no problem taking care of COVID-19 patients”* (NK5).

#### Patients Should Be Treated as if They Were Family Members

The participating nurses perceived all patients as if they were their family members. The nurses asked themselves the question of what they would do if the COVID-19 patient was one of their family members and needed someone to take care of them. Therefore, they treated COVID-19 patients the way they would have liked their family members to be treated. One nurse reported:

“*The motive behind my voluntary work with patients despite the challenges is the idea that each patient could have been one of my family members”* (NH1).

Another participant said, “*I kept saying that these patients have nothing to do with the being infected …he/she could be my father, my mother, my sister…you should consider patients as family members …. I used to work with pregnant women with COVID-19, and I kept thinking that this could have been my wife who was pregnant during these times”* (NK2).

### Ethical Dilemma

The thematic analysis of the interviews showed that the nurses were caught in an ethical dilemma. On the one hand, they felt that they should not be forced to work with COVID-19 patients, and on the other hand, they felt a national and professional sense of commitment to not saying no. Two subthemes emerged from this theme: first, working with COVID-19 patients should Be voluntary and not obligatory, and second, nurses have a community and professional commitment to caring for all patients.

#### Working With COVID-19 Patients Should Be Voluntary and Not Obligatory

Although all of the nurses in this study had voluntarily participated in the care of patients with COVID-19, they nonetheless believed that nurses should not be forced to provide care for COVID-19 patients. They highlighted that nurses could have personal or social factors that placed them and their families at risk. For example, some female nurses could be pregnant, which would place them at high risk, and other nurses could have at-risk family members, such as children or elders. One nurse stated:

“*Some nurses might have serious circumstances that could prevent them from taking care of patients with COVID-19. For example, some nurses might be pregnant, whilst others might have family members with low immunity, and if nurses contract the virus, they might pass it on to their family members. Also, some nurses might have children”* (NH3).

Another participant reported:

“*First, I try to assess the situation and find out why the nurse is refusing to work with COVID-19 patients and whether the reasons are logical or illogical. The nurse might have a logical reason, such as certain family circumstances or psychological or pathological conditions. In these situations, we may accept the nurse's refusal to work”* (NH10).

#### Caring for COVID-19 Patients Is a Community and Professional Commitment

Some of the participating nurses believed that they had a commitment toward their country and their profession to take care of patients with COVID-19. They emphasized that as nurses, they had no choice but to agree to work with COVID-19 patients. One nurse stated:

“*As a nurse, you do not have a choice of whether to work or not …. regardless of whether the disease is infectious or not …stable or not …These are patients and you need to take care of them…. I am a nurse and this is what my job requires”* (NK4).

Another nurse reported:

“*I am one of those nurses who would never say no…I feel that this is a community duty …I was asked, so I went for it”* (NK5).

The nurses in this study found it very difficult to refuse taking care of patients with COVID-19, and they indicated that even if they were to be asked again to take care of COVID-19 patients, they would still say yes. One nurse stated:

“*Even if I were to be asked again, I would not say no. I don't know …I feel it's my professional obligation to not refuse any work …I as* [name of participant] *am not here to be selective in my work and to work only with stable, noninfectious cases…In the end, you as a nurse should provide care not only to stable patients but to all patients, whether they have H1N1 or COVID-19 or AIDS”* (NH10).

### Nurses Are Responsible for Protecting Themselves

As frontline healthcare providers, the participating nurses believed that they needed to protect themselves so as to not contract the virus. Thus, they believed that they were obligated to have sufficient knowledge and to protect themselves appropriately. They considered this to be part of their accountability as nurses. One nurse stated:

“*You have some people in the community who wouldn't forgive you if you caught the virus…They would say that healthcare providers are the ones responsible for the spread of the virus … I am cautious when treating patients and follow precautions …so that I won't be accountable and won't be asked by people”* (NH2).

Some of the interviewed nurses expressed their fear of contracting the virus, as this is perceived as a social stigma. One nurse reported:

“*You know, if I were to get infected, this would be a stigma…People would say that I am responsible for spreading the disease to the world (laugh). Seriously, I felt scared, so I isolated myself. But even so, people wouldn't forgive me if they found out that I had the virus”* (NK5).

Another participant emphasized:

“*At the beginning, we had a few members of healthcare staff who got infected and transmitted the disease to their families. Thus, people are considering them responsible and blame them as if they're the reason* [for the spread of the disease]” (NH4).

## Discussion

Nurses are frontline workers providing care for patients, and they struggle with many ethical challenges when providing patient care. Nurses are often faced with everyday ethical decisions in nursing practice that may seem insignificant but which may be stressful for nurses, as they must face the question of what is right and what is wrong (15). This is particularly the case during these times, given the outbreak of the COVID-19 pandemic around the globe. The present study aimed to explore nurses' ethics in the care of patients with COVID-19, considering the fact that it may become particularly difficult for nurses to offer their care and help during a pandemic like the COVID-19 pandemic.

Nursing practice is guided by a professional code of ethics, which is applicable wherever and whenever nurses are working. The code allows nurses to identify ethical issues and provides guidance on how to take ethical decisions and actions when providing care. In certain circumstances, nurses are allowed to choose not to provide patient care. The safety of nurses and other frontline healthcare workers is a pressing ethical concern, as they are often asked to work under conditions that pose substantial and inadequately understood risks to their overall health and well-being. In addition, nurses may choose not to provide patient care if they lack the support they need to meet their personal and family needs or if they are also worried about the moral, professional, and legal protection when providing nursing care.

During emergencies such as pandemics, nurses may prioritize other aspects of emergencies, such as mitigation, preparedness, response, and recovery, over human rights. However, they can only do so after presenting logical reasons, obtaining consent from concerned authorities, and meeting international standards. Nonetheless, even in such situations, it is the duty of nurses to promote patient health and follow proper protocols to prevent all those involved from oppression (16).

The nurses in the current study reported that it was their duty to care for patients regardless of their medical condition or diagnoses. They expressed that this was part of their professional ethics, which are framed by standards of human rights. Since the nurses were aware that their profession is directed toward the care of patients and the community, they believed that they had a national/community commitment to not refuse to provide care for any patient. In addition, the most common ethical issues among the majority of the nurses in the current study were related to the protection of patients' rights, since this is one of the founding principles of nursing practice. The value of beneficence, professional advocacy, and serving the best interests of patients is emphasized by both national and international nursing standards of ethical behavior.

Patients who suffer from any disease feel anxious and uncertain (17), and this is especially the case with COVID-19, a disease with unclear prognosis and treatment. Such diseases, which are often contagious, may be stigmatized by society, and patients may therefore require support and care. It is mostly nurses who are expected to provide this care and support, regardless of the patient's diagnosis.

The nurses in the current study also shed light on the challenges faced by nurses when the COVID-19 patient is also a family member. It is a challenging experience being a nurse, and caregiver, and taking care of relatives. Nurse family careers actively engage in possibilities to maintain a sense of engaged involvement in the everyday caring for their relatives (18).

Support at the organizational level is of great significance for registered nurses. Nurses should be actively involved in the development and implementation of policies related to the quality of care, especially during exceptional circumstances such as the COVID-19 pandemic. Thus, effective communication between registered nurses and their organizations' management teams is essential. Nurses' capabilities of providing patient care should be acknowledged at all organizational levels, and their concerns should be heard and addressed.

The nurses in the current study had volunteered to work with patients with COVID-19, as they believed that it is part of nurses' responsibility to take care of patients regardless of their diagnoses. Nonetheless, the participating nurses expressed that working with COVID-19 patients should not be obligatory, as some nurses may be incapable of providing the required care in unexpected and unclear circumstances such as the COVID-19 pandemic. For example, some nurses may be sick or may have social obligations such as caring for children or elderly family members. The participating nurses felt that it is the obligation of employers to foster work environments wherein the health and well-being of healthcare professionals are ensured. This may include the provision of immunizations and adequate personal protective equipment, in addition to other operational protocols. Moreover, it is impossible to follow the ethical requirements of clinical practice without adequate staffing. Understaffing and other systematic challenges could impede nurses from performing many of their primary duties, such as maintaining the needs of particular patients and families, alleviating pain, and maintaining their own honesty.

While investigating the reasons behind the event of loss of numerous lives by the breakout of severe acute respiratory syndrome (SARS) in 2003, the researchers working at the University of Toronto Joint Center for Bioethics reported that the healthcare teams were not fully equipped with the knowledge to deal with emergencies and pandemics (19). It was therefore suggested that healthcare institutes should specify beforehand clear guidelines related to dealing with the outbreak of any contagious disease. The researchers also suggested that measures be taken to improve the existing methods of creating awareness among healthcare workers regarding their duties and roles during the outbreak of contagious diseases (19).

A main question that arises during pandemics, such as the COVID-19 pandemic, is whether nurses can refuse to provide patient care in order to protect themselves. Nurses may refuse to provide patient care when this is in the best interest of their personal safety and well-being, as well as their families' (20). In such contexts, the basic right of nurses to protect themselves and their families cannot be overlooked or denied (21–23). On the other hand, there is the view that nurses are professionally responsible for caring for patients regardless of the personal consequences. Therefore, in times such as the COVID-19 pandemic, nurses may find themselves faced with the ethical dilemma of whether to refuse to provide patient care in order to protect themselves or to provide patient care regardless of the consequences this may entail.

The study has a few limitations. A small sample size (*n* = 10) may not represent all nurses working with COVID-19 patients, but this number is sufficient for no new themes to emerge. Also, there was repetition in the information provided and reached saturation. Another limitation of this qualitative study's findings is not intended to be generalized but rather to be used to gain an understanding of the experiences of nurses working with COVID-19 patients.

## Conclusion

Healthcare providers, including nurses, play a significant role during pandemics and other emergencies in facilitating the provision of healthcare and reducing the damage caused by such disasters. Sympathy has been indicated as a nursing ethical value with traits of understanding the needs of patients and their families and providing care based on moral and ethical standards. The standards of practice and ethical codes to be followed by nurses in the provision of healthcare during pandemics or disasters are specified by current laws and agreements. Working in the healthcare sector entails that nurses should think about their ethical responsibilities, challenging duties, and professional and personal values prior to the occurrence of emergencies.

## Data Availability Statement

The raw data supporting the conclusions of this article will be made available by the authors, without undue reservation.

## Ethics Statement

The studies involving human participants were reviewed and approved by This Study was conducted after obtaining the approval of the Institutional Review Board at the Jordan University of Science and Technology. Written informed consent for participation was not required for this study in accordance with the national legislation and the institutional requirements.

## Author Contributions

All authors listed have made a substantial, direct and intellectual contribution to the work, and approved it for publication.

## Conflict of Interest

The authors declare that the research was conducted in the absence of any commercial or financial relationships that could be construed as a potential conflict of interest.
